# Whether Chinese Medicine Have Effect on Halitosis: A Systematic Review and Meta-Analysis

**DOI:** 10.1155/2018/4347378

**Published:** 2018-11-26

**Authors:** Xinyu Wu, Jie Zhang, Yikun Zhou, Ze He, Qiaoyi Cai, Min Nie

**Affiliations:** The State Key Laboratory Breeding Base of Basic Science of Stomatology, Hubei Province & Key Laboratory of Oral Biomedicine (Wuhan University), Ministry of Education, School and Hospital of Stomatology, Wuhan University, Luoyu Road 237, Wuhan 430079, Hubei, China

## Abstract

**Object:**

Halitosis has great adverse impact on personal and social life. There is no strong evidence for the effect of Chinese medicine (CM) and combined Chinese and western medicine (CWM) on halitosis. The aim of the present study is to evaluate the effective rate of CM and CWM on halitosis.

**Materials and Methods:**

Literature search in English and Chinese was conducted in PubMed, Embase, CNKI, CBM, and Wanfang database. Study selection and data collection were conducted. Risks of bias were assessed by the Cochrane tool. Synthesis of results was done by RevMan 5.3. p<0.05 was considered significant difference. Subgroup analysis by classification of halitosis and sensitivity analysis were also conducted.

**Results:**

Seventeen studies were included. The follow-up length ranged from five days to eight weeks. CM had significantly better effect than WM on intraoral halitosis (I^2^ =24%; RR=1.21 (95% CI, 1.04, 1.40), P=0.01) and extraoral halitosis (I^2^ =0; RR=1.39 (95% CI, 1.19, 1.63), P<0.0001). CWM had significantly better effect than WM on intraoral halitosis (I^2^ =0; RR=1.25 (95% CI, 1.16, 1.35), P<0.00001) and extraoral halitosis (I^2^ =0; RR=1.19 (95% CI, 1.08, 1.31), P=0.0004). Subgroup analysis and sensitivity analysis showed insignificant results.

**Conclusion:**

With the limitation of our study, both CM and CWM have significantly better effect on halitosis than WM. More effort should be made to explore long-term effect of CM and CWM on halitosis. This study was registered with the PROSPERO (ID: CRD42018107229).

## 1. Introduction

Halitosis is defined as offensive odor exhaling from oral cavity, the main component of which is volatile sulphur compounds (VSCs) including hydrogen sulfide, dimethyl sulfide, and methyl mercaptan [1]. Halitosis, with a world-wide prevalence rate ranging from 15% to 93% [2–7], is considered as the most disfavoring aspect in personal and social life [8].

Halitosis is classified as genuine halitosis, pseudo-halitosis, and halitophobia [9]. Eighty to ninety percent of genuine halitosis has intraoral sources, including gingivitis, periodontitis, and tongue coating in favor of microorganism [8]. Halitosis with extraoral sources comes from systematic diseases such as nasal inflammation, diabetes mellitus, respiratory, and digestive diseases or medication [9]. Current western medicine (WM) for halitosis mainly includes mechanical methods (periodontal initial treatment, oral prophylaxis, tooth brushing, flossing, and tongue cleaning) and chemical methods (chlorhexidine, essential oil, menthol, chlorine dioxide, and two-phase oil-water rinse) [10]. However, WM mainly diminish the level of VSCs or related anaerobic bacteria thus having drug resistance and side effect on the existing oral microbial ecology [11]. In cases of extraoral health, WM discussed above has little effect on the systematic sources, resulting in relatively high recurrence rate and low patient satisfaction [2]. Besides, there is no way to treat halitosis with unknown sources [9].

Research in halitosis in traditional Chinese medicine (CM) dates back to thousands of years ago. According to ancient books in China, halitosis is defined as rotting smell from mouth and has been treated with acupuncture, moxibustion, and decoction of Chinese herbs. Recently, a number of randomized controlled clinical trials in China reported that orally administrated CM and combined Chinese and western medicine (CWM) have superior effects on halitosis than WM alone. But no synthesis of those results has been made. Accumulating evidence has aroused interest in CM and CWM as alternative methods for halitosis.

The aim of the present study is to evaluate effective rate of CM and CWM versus WM on intraoral and extraoral halitosis. We review randomized controlled clinical trials, of which the intervention is CM or CWM and the control is WM.

## 2. Materials and Methods

### 2.1. Study Design and Registration

This systematic review was written according to the PRISMA list [29]. The protocol has registered in the PROSPERO (ID: CRD42018107229).

### 2.2. Eligible Criteria

Only randomized controlled clinical trials were included in this systematic review. The intervention should be Chinese herbs for mouth rinse or taken orally, combined with western therapy or not. The control should be western therapy. Subjects have either intraoral halitosis or extraoral halitosis. To be diagnosed as intraoral halitosis, subjects should not have systematic disease that could induce oral malodor. Outcome measurements should include effective rate.

Studies were excluded if halitosis was a syndrome of disease and not specially estimated. RCTs in which control group is blank or placebo were also excluded.

### 2.3. Search Strategy

Two individual researchers (XY Wu and J. Zhang) conducted literature search independently and in duplicate. A third researcher (M Nie) was consulted if disagreements occurred. We searched articles in English or Chinese, from database including CNKI, CBM, Wanfang, PubMed, and Embase from 2000.1 to 2018.6.

In CNKI, CBM, and Wanfang, search strategies were (“kouchou” OR “kouqiangyiwei”) AND (“zhongyi” OR “zhongxiyijiehe” OR “zhongyao”). In PubMed and Embase, search strategies were (“halitosis ” OR “bad breath” OR “oral malodor” OR “breath ordor”) AND (“Chinese medicine” OR “combined traditional and Western medicine” OR “chinese herb*∗*”)

### 2.4. Study Selection and Data Collection

Study selection and data collection were conducted by two researchers (XY Wu and J. Zhang), independently and in duplicate. Any disagreement was solved by discussion with a third researcher (M. Nie).

Study selection procedure was conducted according to the inclusion and exclusion criteria. Firstly, all the results in the databases above were gathered together and duplications were discarded. Secondly, Titles and abstracts were scanned. Full-texts were accessed if they might meet our criteria or they were needed for further confirmation. Thirdly, we assessed full-texts and determined the included studies. In this step, reasons for excluding studies were recorded. We also conducted a manual search in the references and citation database of included studies.

After study selection, data for all included studies were extracted. Key information included first author, country, publication year, the number of subjects, criteria for halitosis diagnosis and treatment effect assessment, treatment methods, follow-up length, and outcome measurements. Authors were contacted for missing data if necessary.

### 2.5. Risks of Bias Assessment

The Cochrane tool [30] was used to evaluate risks of bias. According to the tool, selection bias, performance bias, detection bias, attrition bias, and reporting bias were evaluated according to the sequence generation, allocation concealment, blinding, incomplete outcome data, and other potential risks. In this systematic review, risks of bias induced by different halitosis diagnosis criteria included studies were reflected in “Other bias”. Judgements were classified as “high risk of bias”, “unclear risk of bias”, and “low risk of bias”.

### 2.6. Synthesis of Results

Review Manager 5.3 was used to synthesize data. We used dichotomous outcome measures to evaluate effective with 95% CI. p<0.05 was considered statistically significant. The fixed effect model was used when less than four studies were included in a meta-analysis while the random-effects model was used when four studies or more were included. Statistical heterogeneity among the studies was evaluated with the Cochrane Q test and I^2^ statistic.

### 2.7. Subgroup Analysis and Sensitivity Analysis

Subgroup analysis by classification of halitosis was conducted. Sensitivity analysis was conducted by excluding studies in which no criteria for halitosis diagnosis were mentioned. When studies in one group had ten studies or more, we evaluated the possibility of publication bias by a funnel plot of the mean differences for asymmetry.

## 3. Results

### 3.1. Study Selection

**Figure 1:**
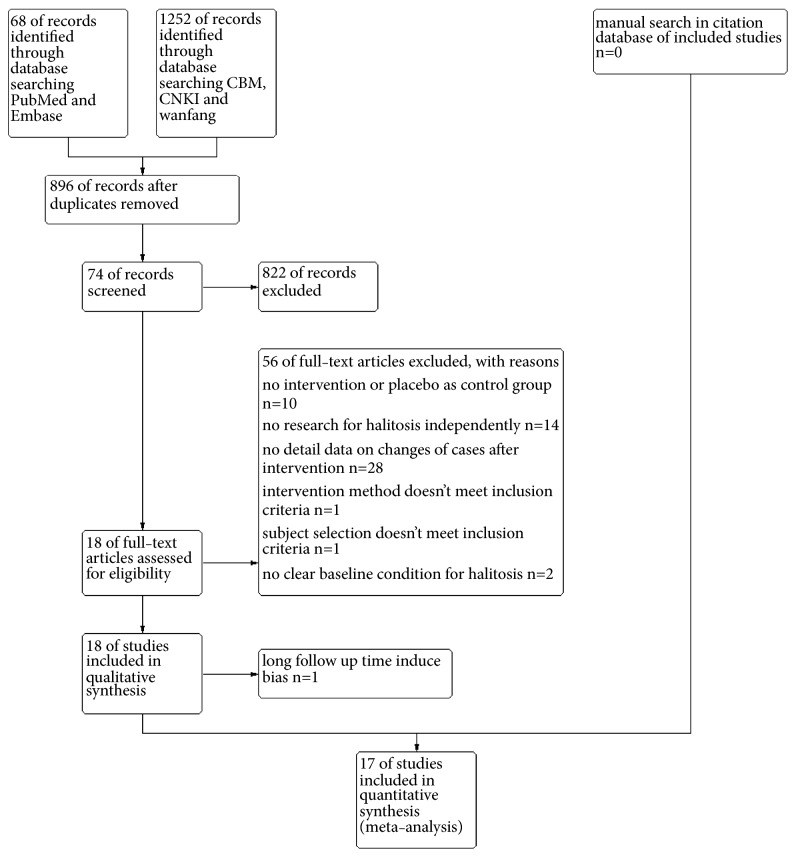
Flow diagram for study selection.

We got 21, 47, 361, 369, and 522 results from PubMed, Embase, CNKI, Wanfang, and CBM, respectively. After discarding duplications, 896 results remained. Titles and abstracts were scanned and 822 articles were excluded. For the remaining 74 articles, we assessed full-texts and excluded 56 articles. Reasons for exclusion are listed in Figure 1. Besides, one study [31] was excluded from meta-analysis for longer follow-up length (one year) than others. Seventeen studies were included in the final quantitative synthesis. Details are listed in Figure 1.

### 3.2. Study Characteristics

All the 17 articles were randomized controlled clinical trials in Chinese. Intervention includes CM and CWM. For the effect of CM on halitosis, six studies were included [12–17] with a follow-up length ranging from five days to eight weeks. For the effect of CWM on halitosis, 11 studies were included [18–28], with a follow-up length ranging from one week to one month. Intraoral halitosis mainly originated from gingivitis, periodontitis, and poor oral care. Extraoral halitosis mainly originated from systematic diseases such as gastritis, constipation, and children amygdalitis. Criteria for halitosis diagnosis and treatment effect assessment were different among studies. Details are listed in Table 1.

### 3.3. Risks of Bias Assessment

**Figure 2:**
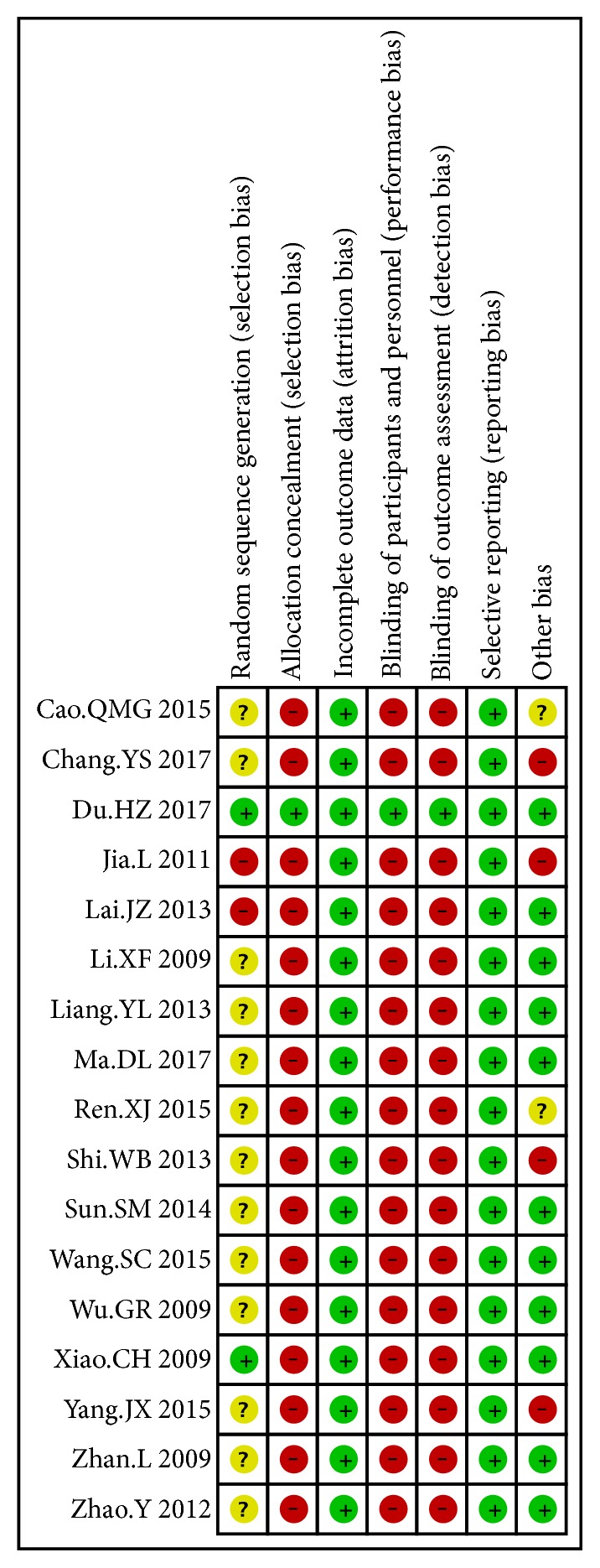
Risk of bias item in each included study.

**Figure 3:**
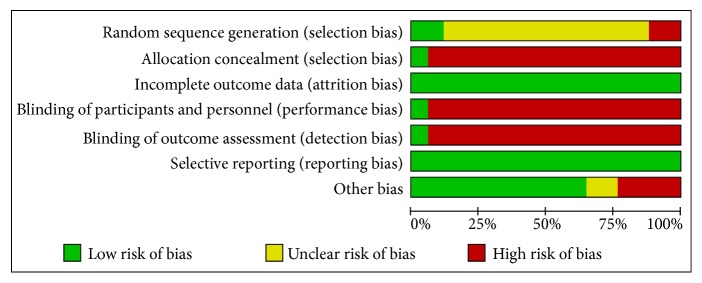
Risk of bias item among included studies.

For randomization, three studies described the method and were considered appropriate. Thirteen studies were described as randomized without describing the methods. And three studies did not mention method of randomization. Only one study described methods of allocation concealment and double blinding appropriately. All studies fully reported outcomes described in methods. No withdrawal or dropout was reported. Other risks of bias were assessed regarding different halitosis diagnosis criteria. Four studies did not mention any method for halitosis diagnosis, considered as high risks of bias. Two studies mentioned an organoleptic test without describing evaluation scale, considered as unclear risks of bias. All included studies showed high risks of bias in at least one domain. (Figures 2 and 3)

### 3.4. Results of Individual Studies

**Figure 4:**
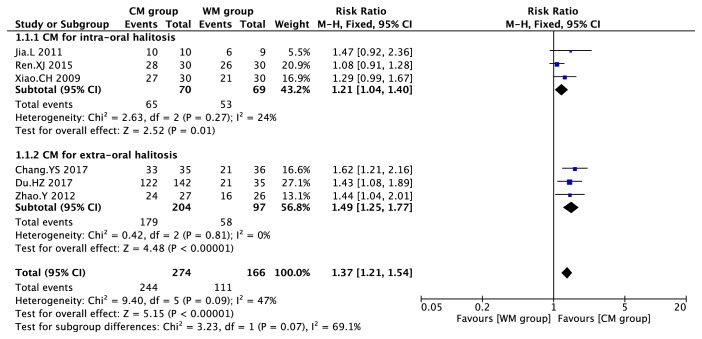
Forest plot for CM on halitosis.

For effective rate of CM on halitosis (Figure 4), CM had significantly better effect than WM on intraoral halitosis (I^2^ =24%; RR=1.21 (95% CI, 1.04, 1.40), P=0.01) and extraoral halitosis (I^2^ =0; RR=1.39 (95% CI, 1.19, 1.63), P<0.0001). Subgroup analysis showed insignificant result (P=0.07, I^2^ =69.1%).

**Figure 5:**
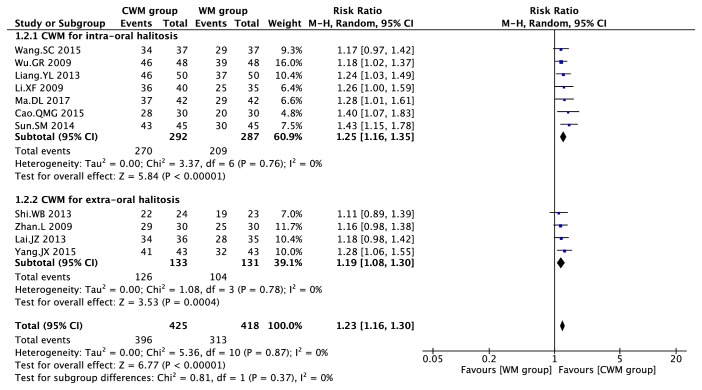
Forest plot for CWM on halitosis.

For effective rate of CWM on halitosis (Figure 5), CWM had significantly better effect than WM on intraoral halitosis (I^2^ =0; RR=1.25 (95% CI, 1.16, 1.35), P<0.00001) and extraoral halitosis (I^2^ =0; RR=1.19 (95% CI, 1.08, 1.31), P=0.0004). Subgroup analysis showed insignificant result (P=0.37, I^2^ =0).

### 3.5. Additional Analysis

**Figure 6:**
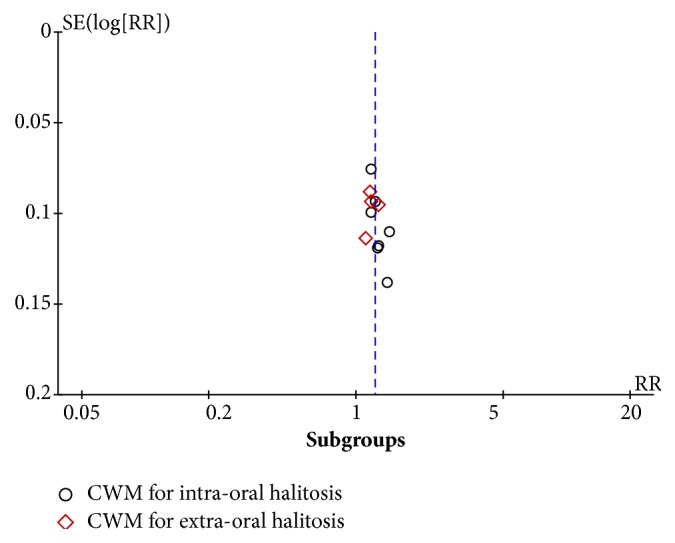
Funnel plot of included studies on CWM.

For sensitivity analysis, four studies were excluded in which no criteria for halitosis diagnosis were mentioned. The effect of CM on intraoral halitosis (I^2^ =29%; RR=1.17 (95% CI, 1.00, 1.36), P=0.04) and extraoral halitosis (I^2^ =0; RR=1.44 (95% CI, 1.16, 1.78), P=0.001) was significantly better than that of WM. The effect of CWM on intraoral halitosis (I^2^ =0; RR=1.25 (95% CI, 1.16, 1.35), P<0.00001) and extraoral halitosis (I^2^ =0; RR=1.17 (95% CI, 1.03, 1.33), P=0.01) was significantly better than that of WM. Sensitivity analysis presented robust results. A funnel plot was made for included studies on CWM on halitosis versus WM (Figure 6).

## 4. Discussion

To the best of our knowledge, this is the first study to synthesize the results of the effect of CM and CWM on halitosis. With the limitation of follow-up length, both CM and CWM have significantly better effect on halitosis than WM.

Better effect of both CM and CWM on halitosis was in accordance with previous studies. An in vivo study [32] reported that chlorhexidine mouth rinse, containing pericarp extract of* Garcinia mangostana* L., significantly reduced VSCs level in gingivitis patients. Li MY et al. [33] reported that toothpaste mixed with Chinese herb extract showed better in vitro inhibition effect on VSCs genesis anaerobic bacteria than most other toothpaste on market. A four-week-period RCT [34] reported that Chinese herb Hyangsa-Pyeongwi san could alleviate halitosis and increase quality of life in functional dyspepsia patients and the effect only lasted for four weeks.

In CM theory, halitosis originates from retention of damp, heat or fire in mouth, stomach, liver, or spleen, all of which are interconnective as an entirety. So, CM treatment concentrates on a balance of the whole body rather than a certain organ. For example, Chinese herbs with “cold” properties, such as* Coptis chinensis*,* Scutellaria baicalensis*,* Lonicera japonica*, the root of red-rooted salvia, and rhizoma zingiberis, could clear away the damp, heat, and fire in body and cure halitosis [35]. Modern pharmacology explained the mechanisms of these herbs.* Coptis chinensis* has properties including antibacterial, antitoxin, antiulcer, and reducing gastric acid [36]. It decreased level of inflammatory cytokines such as VEGF and TNF in arthritis mouse serum and regulated the cell proliferation, differentiation, and apoptosis related genes to alleviate inflammation [37, 38]. The extraction of* Coptis chinensis* could also inhibit urease to anti-*H. pylori*, the main inducer of gastritis-related halitosis [39–41]. Scutellaria baicalensis has effects on iNOS, COX2, NF*κ*B, and inflammatory cytokines like IL-1*β*, IL-2, IL-6, IL-12, and TNF-*α* [42–44].* Lonicera japonica* had strong effects on VSCs and bacteriostasis functions on halitosis related anaerobic bacteria [45, 46].

In this study, CM shows better effect on extraoral halitosis while CWM shows better effect on intraoral halitosis, although the difference was not significant. According to the CM theory of “entirety”, Chinese herbs should be admitted orally to be absorbed by body to exert influence. On the contrary, periodontal treatment in WM could reduce oral VSCs and related anaerobic bacteria immediately. So, the effect by CM was limited by a short follow-up length in this study. Better effect of CWM on intraoral halitosis suggested that CM could strengthen the effect of WM and, in turn, CM acted better on the basis of WM. CM and WM benefit from each other and combining them together lead to even superior results.

Different criteria for halitosis diagnosis and treatment effect assessment were used among included studies. However, sensitivity analysis showed no significant result. The gold standard for diagnosis of halitosis is organoleptic measurement [47], which is subjective and inconsistent. Furthermore, it is hard to grade the severity of halitosis with clear boundaries. This partly explains the low inheterogeneity among included studies despite different evaluation scales being used. Future studies should use quantifiable outcome measures, such as component in breath or saliva, to make their results reproducible and objective.

The protocol of this study has registered in the PROSPERO to ensure a qualified methodology. The whole procedure was conducted according to the PRISMA list. We have done comprehensive literature search and covered nearly all available studies. Subgroup and sensitivity analysis was made to ensure the reliability of results. However, all studies included were in Chinese and scored high risks of bias in at least one domain. In one study [13] CM mouth rinse was used in treating intraoral halitosis while in others CM was taken orally. Only studies with short follow-up length (no longer than 8 weeks) were included. Till now evidence was still insufficient for long-term effect of CM and CWM on halitosis.

## 5. Conclusion

With the limitation of our study, both CM and CWM have significantly better effect on halitosis versus WM. Combining Chinese medicine and western medicine has quicker and stronger effect on halitosis in short term. More effort should be made on long-term effect of CM and CWM on halitosis.

## Figures and Tables

**Table 1:** Study characteristics for included studies.

	Subjects (INT/CON)	Halitosis	Intervention	Control	Follow-up length	Diagnosis*∗*	Treatment effect*∗∗*
Xiao et al.(2009)[12]	30/30	intra-oral	CM taken orally	PIT	10d	A	E
Ren et al.(2015)[13]	30/30	intra-oral	CM mouth rinse	2.5% sodium bicarbonate solution mouth rinse	10d	C	F
Jia et al.(2011)[14]	10/9	intra-oral	CM mouth rinse	PIT	1m	D	F
Zhao et al.(2012)[15]	27/26	extra-oral	CM taken orally	WM taken orally	8w	B	E
Chang et al(2017)[16]	35/36	extra-oral	CM taken orally	WM taken orally	1m	D	E
Du et al (2017)[17]	213/104	extra-oral	CM taken orally	WM taken orally	5d	B	E
Liang et al.(2013)[18]	50/50	intra-oral	CM taken orally, PIT	PIT	10d	A	E
Wu et al.(2009)[19]	48/48	intra-oral	CM taken orally, PIT	PIT	2w	A	E
Wang et al.(2015)[20]	37/37	intra-oral	CM taken orally, PIT	PIT	10d	A	E
Ma et al.(2017)[21]	42/42	intra-oral	CM taken orally, PIT	PIT	10d	A	E
Li et al.(2009)[22]	40/35	intra-oral	CM taken orally, PIT	PIT	10d	A	E
Sun et al.(2014)[23]	25/23	intra-oral	CM taken orally, PIT	PIT	10d	A	F
Cao et al.(2015)[24]	30/30	intra-oral	CM taken orally, PIT	PIT	1w	C	F
Zhan et al.(2009)[25]	30/30	extra-oral	CM taken orally, WM taken orally	WM taken orally	1m	A	E
Yang et al.(2015)[26]	43/43	extra-oral	CM taken orally, surgery plus WM taken orally	Surgery, WM taken orally	3w	D	F
Shi et al.(2013)[27]	24/23	extra-oral	CM taken orally, WM taken orally	WM taken orally	1w	D	F
Lai et al.(2013)[28]	36/35	extra-oral	CM taken orally, WM taken orally	WM taken orally	1m	A	E

INT/CON=intervention group/control group; PIT=periodontal initial therapy.

*∗* Criteria for halitosis diagnosis. A, organoleptic measurement and Rosenberg scale. In an organoleptic test, the patient takes deep breath by nose, hold it for a while, and exhale by mouth. An examiner standing 20cm away from the patient assess and classify the severity of bad odor as 0 to 5 points (Rosenberg scale, 0: no odor, 1: barely noticeable, 2: slight but clearly noticeable, 3: moderate, 4: strong, and 5: extremely strong). Halitosis was diagnosed if a patient scored 2 points or higher. B, CM symptom rating scale (0: no odor, 1: self-sensed odor, 2: others could smell bad odor, and 3: sever odor that keeps others away from patient). C, olfaction diagnosis but no specific method was described. D, not mentioned. Halitosis was diagnosed if a patient scored 1 point or higher.

*∗∗* Criteria for treatment effect. E, bad odor alleviated and scores reduced by no less than one point after treatment. F. bad odor alleviated or disappeared after treatment.

## Data Availability

No additional data is available.
